# Linked Data Paves the Way to Improved Health Care

**DOI:** 10.23889/ijpds.v1i1.306

**Published:** 2017-04-19

**Authors:** Michael Gaucher, Britta Nielsen, Katerina Gapanenko

**Affiliations:** Canadian Institute for Health Information

## Objectives

Our organization links data from health care organizations and health ministries across Canada to create new knowledge. The new knowledge supports system and policy-decision makers in answering questions about the health or healthcare of population groups or healthcare sectors. This presentation provides an overview of recent analytical products that incorporate a variety of approaches to linking data.

## Method

Key success factors in data linkages are the use of standards and robust methodologies. Standardized data can be linked at the individual level across hospital, long-term care, home care and community settings. Expanding knowledge about a specific sector can be achieved by linking physician billing, drug, and financial data. Linking clinical and financial data at the person and/or organization level provides insight into the cost of providing services, laying the foundation for examining efficiency and value for money. Data may also be used in non-traditional ways, for example, using payment data linked to clinical data to better understand how services are provided in various settings and to identify best practices.

## Results

Results from these studies demonstrate how linked data creates new knowledge:

1. Hospital, emergency, and drug data were linked to show the number of emergency visits, hospitalizations, and the use of psychotropic medications by children and youth with mental health disorders are increasing, especially for those with mood and anxiety disorders living in urban areas.
2. Physician billing data was linked with hospital and emergency data to show that increased continuity of care with a family physician is associated with reductions in hospitalizations for people with chronic diseases and reductions in ED visits for people with conditions better managed in primary health care settings.
3. Drug data was linked with clinical assessment (InterRai) data to show that a large proportion of seniors living in long-term care exhibiting severe aggression were not being treated with antipsychotics, suggesting non-drug alternatives were often considered
4. Financial data was linked with hospital clinical data to calculate an efficiency indicator that can be used at a system level to examine variation, and by individual managers to identify areas for further investigation and manage trends in spending

## Conclusion

This presentation demonstrates how new knowledge created using linked data can inform decision and policy makers, and lead to action within the health system. Several examples are presented to illustrate the approach and types of data being linked.

